# Adverse drug reactions of montelukast in children and adults

**DOI:** 10.1002/prp2.341

**Published:** 2017-09-20

**Authors:** Meindina G. Haarman, Florence van Hunsel, Tjalling W. de Vries

**Affiliations:** ^1^ Department of Pediatric Cardiology Center for Congenital Heart Diseases Beatrix Children's Hospital University Medical Center Groningen The Netherlands; ^2^ Netherlands Pharmacovigilance Center Lareb Den Bosch The Netherlands; ^3^ Department of Pediatrics Medical Center Leeuwarden Leeuwarden The Netherlands

**Keywords:** Asthma, drug safety, therapeutic drug monitoring

## Abstract

Montelukast, a selective leukotriene receptor antagonist, is recommended in guidelines for the treatment of asthma in both children and adults. However, its effectiveness is debated, and recent studies have reported several adverse events such as neuropsychiatric disorders and allergic granulomatous angiitis. This study aims to obtain more insight into the safety profile of montelukast and to provide prescribing physicians with an overview of relevant adverse drug reactions in both children and adults. We retrospectively studied all adverse drug reactions on montelukast in children and adults reported to the Netherlands Pharmacovigilance Center Lareb and the WHO Global database, VigiBase^®^ until 2016. Depression was reported most frequently in the whole population to the global database VigiBase^®^ (reporting odds ratio (ROR) 6.93; 95% CI: 6.5–7.4). In the VigiBase^®^, aggression was reported the most in children (ROR, 29.77; 95% CI: 27.5–32.2). Headaches were reported the most frequently to the Dutch database (ROR, 2.26; 95% CI: 1.61–3.19). Furthermore, nightmares are often reported for both children and adults to the Dutch and the global database. Eight patients with allergic granulomatous angiitis were reported to the Dutch database and 563 patients in the VigiBase^®^. These data demonstrate that montelukast is associated with neuropsychiatric adverse drug reactions such as depression and aggression. Especially in children nightmares are reported frequently. Allergic granulomatous angiitis is also reported, a causal relationship has not been established.

**Abbreviations**
ADR: adverse drug reaction
ATC: anatomical and therapeutic chemical classification
ICSR: individual case safety report
LTRA: leukotriene receptor antagonist
MedDRA: Medical Dictionary for Regulatory Activities
ROR: reporting odds ratio
SmPC: summary of product characteristics
UMC: Uppsala Monitoring Center
WHO: World Health Organization

## Introduction

Montelukast is a selective leukotriene receptor antagonist (LTRA) and is prescribed in both children and adults for, that is, the maintenance treatment of asthma and allergic rhinitis. Although the effectiveness is debated, (Hon et al. 2014; Brodlie et al. 2015, 2016) guidelines recommend montelukast for treatment of asthma (British Thoracic Society; Scottish Intercollegiate Guidelines Network, 2014). The most common adverse events in adults according to the summary of product characteristics (SmPC) are upper airway infections (in >10% of all users) fever, rash, nausea, vomiting, diarrhea, and elevated levels of liver enzymes (Dutch Farmacotherapeutic Compass, 2016). Most common adverse events in children (1–10% of all users) according to the SmPC are headaches, abdominal pain, rash, thirst, hyperkinesia, asthma, and eczema (Dutch Children's Formulary, 2016). Recent studies have also reported adverse events such as sleeping disorders and psychiatric disorders (Calapai et al. 2014). In addition, allergic granulomatous angiitis (Churg‐Strauss syndrome) may also be associated with the use of montelukast (Calapai et al. 2014).

The aim of this study is to obtain more insight into the safety profile of montelukast in daily practice to provide prescribing physicians with an overview of relevant adverse drug reactions (ADRs) in children and adults. We therefore studied the reports of ADRs associated with montelukast in the Dutch spontaneous reporting database of the Netherlands Pharmacovigilance Center Lareb and the WHO Global Individual Case Safety Report (ICSR) database VigiBase^®^, maintained by the Uppsala Monitoring Center (UMC) in Sweden.

## Materials and methods

We retrospectively examined all ADRs on montelukast (Anatomical and Therapeutic Chemical classification (ATC) code R03DC03) in children aged 0–18 years and adults aged 19 years and older reported to the Netherlands Pharmacovigilance Center Lareb and the WHO Global ICSR database, VigiBase^®^ until 2016.

The reports in the Dutch spontaneous database (until 13‐07‐2016) were coded with the Medical Dictionary for Regulatory Activities (MedDRA^®^) and individually assessed for causality by trained assessors.

For the Netherlands Pharmacovigilance Center Lareb, data, including suspect drug, co‐medication, age and sex of the patient, and the suspected ADR, were extracted from the database. Reported ADRs were classified into the categories “non‐serious” and “serious” based on international criteria. The latter included fatal outcome, life‐threatening, requiring (prolongation of) hospitalisation, resulting in significant disability/incapacity, and other medically important conditions. All other ADRs were classified as nonserious (European Medicines Agency, 2009).

We report the serious and most often reported ADRs. In selected cases, we reassessed the causality of the reported drug reactions based on the Naranjo score. Moreover, the reporting odds ratio (ROR) was calculated for selected associations.

The Naranjo score is a quantitative method for determining the likelihood that an ADR is due to the drug (Naranjo et al. 1981). The ROR compares the rate of reporting a specific adverse effect in a drug with the rate of reporting the same adverse effect in all other drugs. The ROR is calculated by the following division: the numerator is the number of cases in which montelukast was used and a specific ADR was reported divided by the number of cases using montelukast in which this ADR was not reported; the denominator is the number of pediatric and adult cases using other suspected drugs, reporting a specific ADR divided by the number of cases using other suspected drugs without reporting that specific ADR. It is expressed as a point estimate with corresponding 95% confidence intervals (95% CIs). Furthermore, at least three reports have to be present in the database to compute a reliable ROR (Rothman et al. 2004). We calculated RORs for ADRs associated with montelukast based on the whole database and a separate ROR restricted to children <19 years of age. The ROR offers insight into disproportionality of an association, not into causality.

For the global database VigiBase^®^, we obtained numbers of reports and disproportionality (ROR) per reported association, both in adults and children through VigiLyze^®^, which is a search and analysis tool (available to member countries of the WHO Program for International Drug Monitoring).

Because no patients were involved, we did not ask the Institutional Review Board for approval.

## Results

### Netherlands pharmacovigilance center Lareb

In the Dutch spontaneous reporting database, 331 reports on ADRs after montelukast were present of which 124 (37.5%) were reported in men and 203 (61.3%) in women. In 4 cases (1.2%), the gender was unknown. In almost a third (107; 32.3%), the reports concerned individuals aged between 0 and 18 years and in 214 cases (64.7%), adults aged 19 years and older. In 10 cases of ADRs (3%) age was not reported. Of all reports, 45 (13.6%) were reported as serious (Table 1). There were two deaths: a 20‐year‐old woman with pulmonary embolism with a doubtful relation between the use of montelukast and the reaction, and a woman of unknown age with renal failure with a possible relation between the use of montelukast and the reaction. In 26 cases, the ADR led to hospitalisation; 10 patients recovered completely, seven patients have not yet recovered, three patients did not recover, and the recovery status of six patients was not known; none of them died. Reasons for hospitalisation were epilepsy, chest pain, insomnia, movement disorder, toxic skin eruption, neurological disorder, vasculitis, anesthesia, urticaria, gastrointestinal tract bleeding, abnormal liver function test, angioedema, general health deterioration, membranous lipodystrophy, eosinophilia, coughing, and anaphylactic reaction. In 16 patients, the adverse event was called serious because of angioedema, hypersensitivity, fatigue, epilepsy, aggression, pain in extremity, immune system disorder, confusional state, hemorrhage, abnormal dreams, excoriation, eosinophil count increased, and abdominal pain. In one patient, the adverse event was called serious but the report was on an accidental overdose.

**Table 1:** Adverse drug reactions (ADRs) after montelukast reported to the Netherlands Pharmacovigilance Center Lareb and deemed serious, for example, leading to death, hospitalisation, or life threatening condition

Adverse drug reaction	Comments	Relation with montelukast according to the Naranjo score (Naranjo et al. 1981)
Death	20‐year‐old woman with pulmonary embolism Woman of unknown age with renal failure.	
Allergic granulomatous angiitis		
Angioedema	4‐year‐old girl with concomitant use of pulmicort and foradil. Patient was treated with tavergil and prednisone. Montelukast was discontinued. Angioedema disappeared.	Possible
Malaise	45‐year‐old woman with concomitant use of 8 other medicines. Montelukast was discontinued and she recovered.	Possible
Epilepsy	9‐year‐old boy with no concomitant use of other medicines. Montelukast was discontinued and he recovered.	Possible
Chest pain	Man of unknown age with concomitant use of seretide. Montelukast was discontinued and he recovered.	Possible
Hallucination	13‐year‐old boy with no concomitant use of medicines. Montelukast was discontinued and he recovered.	Possible
Myalgia	47‐year‐old man with concomitant use of 5 other medicines. Montelukast was discontinued and he recovered.	Possible
Eosinophilia	66‐year‐old woman with concomitant use of seretide. Montelukast was discontinued and she recovered slowly.	Probable
Nightmare/somnambulism	72‐year‐old woman with concomitant use of 9 other medicines. Montelukast was discontinued and she recovered.	Probable
Chest discomfort	Woman of unknown age with concomitant use of 5 other medicines. Further information unknown Woman of unknown age with concomitant use of 3 other medicines. Further information unknown.	Possible Possible
Anaphylactic reaction	13‐year‐old boy with concomitant use of 4 other medicines. Montelukast was discontinued and he recovered.	Probable

Of the 45 patients with serious adverse events, eight patients had allergic granulomatous angiitis of which six patients were hospitalised; all patients survived. Their characteristics are presented in Table 2.

**Table 2:** Characteristics of six patients hospitalised with allergic granulomatous angiitis reported to the Netherlands Pharmacovigilance Center Lareb

Description	Latency	Concomitant medicine use	Action and outcome	Relation with Montelukast
33‐year‐old woman with a history of asthma	6 months	Doxycycline, prednisone, flixonase	Patient was treated with high dose oral prednisone; not yet recovered at the time of reporting.	Possible
53‐year‐old man with an unknown history	5 years	Flixonase, seretide	Montelukast has been Withdrawn; patient has not recovered at the time of reporting.	Doubtful
55‐year‐old woman with a history of asthma	8 months	Cetirizine, seretide, flixonase	Montelukast has been withdrawn; patient recovered after treatment with prednisone.	Possible
75‐year‐old woman with a history of asthma	7 days	Phenprocoumon, furosemide, digoxine, isosorbide mononitrate, carvedilol, ramipril, omeprazole, tiotropium	Montelukast has been withdrawn; patient was treated with prednisone and is recovering.	Possible
59‐year‐old woman with a history of asthma, rhinitis, and bronchiectasis	3 months	Mometasone, tiotropium, ciclesonide, beclometasone/formoterol	Montelukast has been withdrawn, and the patient is treated with prednisone. She is recovering.	Probable
75‐year‐old woman with a history of asthma	Unknown	Phenprocoumon, omeprazole, calcium carbonate, digoxine, furosemide, ramipril, isosorbide mononitrate, beclometasone/formoterol, tiotropium, cardvedilol	Montelukast has been withdrawn; patient recovered.	Possible

Disclaimer: This publication contains information obtained from UMC through https://vigilyze.who-umc.org (restricted access), accessed at 03‐11‐2016. The information derives from a variety of sources, and the likelihood that the suspected adverse reaction is drug‐related is not the same in all cases. The information provided in this article does not represent the opinion of the World Health Organization. For more information, see http://www.who-umc.org/graphics/25300.pdf

### WHO global ICSR database, VigiBase^®^

In the global spontaneous reporting database, 17,723 reports on ADRs after montelukast were present of which 6960 (39.3%) were reported in men and 9732 (54.9%) in women. In 1031 cases (5.8%), the gender was unknown. Approximately a third (5743; 32.4%) of the reports concerned individuals aged 0–18 years old. Additionally, age was not reported in 3,665 cases of ADRs (20.7%).

### Most common adverse events Netherlands Pharmacovigilance Center Lareb and WHO global ICSR database, VigiBase^®^

Table 3 depicts the most frequent ADRs with RORs for all cases and for individuals under <19 years of age reported to both the Netherlands Pharmacovigilance Center Lareb and the WHO Global ICSR database, VigiBase^®^.

**Table 3:** Most common adverse drug reactions (ADRs) after montelukast reported to the Netherlands Pharmacovigilance Centre Lareb and the WHO Global ICSR database VigiBase^®^

Adverse drug reaction	Total number of reports at VigiBase^®^	ROR^a^ VigiBase^®^ (95% CI)	Number of reports in children <19 year at VigiBase^®^	ROR^a^ VigiBase^®^ in children <19y (95% CI)	Total number of reports at Lareb	ROR^a^ Lareb (95% CI)	Number of reports in children <19 year at Lareb	ROR^a^ Lareb in children <19 year (95% CI)
Depression	1188	6.93 (6.54–7.36)	493	20.52 (18.65–22.58)	5	1.91 (0.79–4.62)	–	–
Headache	1128	1.85 (1.75–1.97)	371	1.91 (1.72–2.12)	37	2.26 (1.61–3.19)	17	3.18 (2.66–3.70)
Aggression	1101	24.99 (23.49–26.59)	808	29.77 (27.54–32.18)	11	9.27 (5.06–16.99)	7	12.02 (11.24–12.80)
Suicidal ideation	1047	20.43 (19.18–21.76)	495	38.27 (34.68–42.22)	1	–	–	–
Insomnia	1020	5.08 (4.77–5.41)	417	11.15 (10.07–12.35)	15	3.45 (2.05–5.81)	7	4.60 (3.83–5.38)
Anxiety	948	5.11 (4.79–5.46)	468	16.99 (15.41–18.72)	6	2.79 (1.24–6.26)	2	–
Abnormal behavior	892	34.05 (31.79–36.46)	643	17.64 (15.99–19.46)	7	12.02 (5.64–25.61)	7	8.56 (7.79–9.34)
Nightmares	749	22.48 (20.87–24.21)	448	78.04 (69.95–87.07)	25	19.29 (12.75–29.17)	13	56.72 (56.09–57.35)
Dyspnea	649	1.30 (1.20–1.41)	120	1.14 (0.95–1.36)	13	1.47 (0.84–2.56)	–	—
Rash	540	0.65 (0.59–0.71)	161	0.31 (0.26–0.36)	17	1.77 (1.09–2.89)	7	1.28 (0.51–2.05)
Abdominal pain	511	1.81 (1.66–1.98)	222	2.24 (1.95–2.56)	15	2.24 (1.33–3.77)	8	3.67 (2.95–4.40)
Dizziness	541	0.89 (0.82–0.97)	97	0.72 (0.59–0.88)	12	0.94 (0.53–1.68)	–	–
Myalgia	352	1.66 (1.49–1.84)	58	1.57 (1.21–2.03)	12	1.26 (0.71–2.25)	–	–
Muscle spasms	291	2.44 (2.17–2.74)	57	3.98 (3.06–5.17)	10	2.87 (1.53–5.40)	–	–
Nausea	557	0.61 (0.56–0.66)	104	0.56 (0.46–0.68)	10	0.65 (0.35–1.23)	4	1.17 (0.16–2.17)

Disclaimer: This publication contains information obtained from UMC through https://vigilyze.who-umc.org (restricted access), accessed at 03‐11‐2016. The information comes from a variety of sources, and the likelihood that the suspected adverse reaction is drug‐related is not the same in all cases, The information shown in this article does not represent the opinion of the World Health Organization. For more information see http://www.who-umc.org/graphics/25300.pdf

a ROR computed when more than two cases. The numerator is the number of cases in which montelukast was used and a specific ADR was reported divided by the number of cases using montelukast in which this ADR was not reported. The denominator is the number of pediatric cases using other suspected drugs reporting a specific ADR divided by the number of pediatric cases using other suspected drugs without reporting that specific ADR. The ROR was calculated for the entire group as well as for children.

As can be observed, depression was reported most frequently in the whole population to the global database, VigiBase^®^. The ROR is 6.93 (95% CI: 6.5–7.4). In the VigiBase^®^, aggression was reported the most in children <19 years of age. The ROR in children is 29.77 (95% CI: 27.5–32.2).

The highest RORs were found for aggression (24.99; 95% CI: 23.5–26.6), suicidal ideation (20.4; 95% CI: 19–22), abnormal behavior (34.05; 95% CI: 31.8–36.5), and nightmares (22.46; 95% CI: 20.9–24.2).

Other common ADRs in the whole population were headaches (ROR 1.85; 95% CI: 1.75–1.970), insomnia (5.08; 95% CI 4.8–5.4), anxiety (5.11; 95% CI: 4.8–5.5), dyspnea (1.30; 95% CI: 1.20–1.41), dizziness (0.89; 95% CI: 0.82–0.97), myalgia (1.66; 95% CI: 1.49–1.84), and muscle spasms (2.44; 95% CI: 2.17–2.74).

Headaches were most frequently reported to the Dutch database for both the whole population and children. The RORs were 2.26 (95% CI: 1.61–3.19) and 3.18 (95% CI: 2.66–3.70), respectively. Other common ADRs in the whole population were aggression, insomnia, anxiety, abnormal behavior, dyspnea, rash, abdominal pain, and muscle spasms. The RORs and 95% CIs for these ADRs can be found in Table 3.

To the Dutch and the global database, nightmares were reported frequently for both children and adults aged 19 years and older. For VigiBase^®^, the RORs were 22.48 (95% CI: 20.8–24.2) and 78.04 (95% CI: 70.0–87.1) for the whole population and children aged <19 years, respectively. For Lareb, the RORs were 19.29 (95% CI: 12.8–29.2) for all cases and 56.72 (95% CI: 56.1–57.4) for individuals under 19 years of age.

In the VigiBase^®^, allergic granulomatous angiitis was reported in 563 patients.

## Discussion

In this study, we found several reported adverse drug events that were deemed serious, and we saw a high number of patients with allergic granulomatous angiitis in both the Dutch and the global database. Most of all, we found a high number of patients with neuropsychiatric adverse effects.

A fatal outcome was reported in two reported adverse drug events in the Netherlands Pharmacovigilance Center database. One patient, a 20‐year‐old woman, died after pulmonary embolism, and another woman of unknown age died due to renal failure. The relation between montelukast and the adverse event in the first patient was considered doubtful, and in the second patient, the relation between the use of montelukast and the reaction was possible. However, due to the limited information in both reported cases, we cannot confirm that these adverse events are caused by the use of montelukast. As far as we could establish, there are no other known cases reported in the literature nor were pulmonary embolism and renal failure found in the VigiBase^®^. Furthermore, we could not detect a specific pattern in the other reports of serious ADRs.

Allergic granulomatous angiitis was reported in eight patients in the Netherlands Pharmacovigilance Center Lareb and in 563 patients in the WHO Global ICSR database, VigiBase^®^. Allergic granulomatous angiitis is a rare disease, and it is stated that allergic granulomatous angiitis will occur in <0.01% of patients treated with montelukast (Dutch Farmacotherapeutic Compass, 2016; Dutch Children's Formulary, 2016). Former studies have revealed that the association between montelukast and allergic granulomatous angiitis is somewhat doubtful. To illustrate, Calapai et al. (2014) argued that most of the patients treated with montelukast who developed symptoms of allergic granulomatous angiitis were also receiving other medications such as corticosteroids or salbutamol, making the relationship with montelukast uncertain. Moreover, all patients with allergic granulomatous angiitis only exhibited symptoms after montelukast had been administered, yet some patients had a decrease in the intake of oral corticosteroids concomitantly. This means that the disease could be masked by the use of corticosteroids and the patient already had angiitis. However, it has been reported that the symptoms of allergic granulomatous angiitis disappeared in some patients after withdrawing montelukast. This can be seen regarded as an argument for a causal relationship.

In an earlier study, it was found that patients treated with montelukast had a 4.5‐fold higher risk of allergic granulomatous angiitis onset within 3 months. Nonetheless, the authors questioned a causal relationship because there could be confounding by a general escalation of asthma therapy before allergic granulomatous angiitis onset (Hauser et al. 2008). We could not find animal studies in which a relation between allergic granulomatous angiitis was established. Further prospective studies in larger patient populations are needed to discern the exact relation between montelukast and the occurrence of allergic granulomatous angiitis. Until then, patients treated with montelukast should be followed to detect signs and symptoms of allergic granulomatous angiitis.

Former studies have provided contradictory reports on neuropsychiatric adverse events in montelukast users. In 2009, the US Food and Drug Administration mandated a label change for montelukast and other leukotriene receptor antagonists to include neuropsychiatric adverse events (e.g., depression and suicidality) as a precaution (FDA, 2017). Nonetheless, Ali could not establish a significant association between montelukast and neuropsychiatric events in children with asthma (Ali et al. 2015).

Our data indicate that neuropsychiatric symptoms, such as depression, aggression, suicidal ideation, abnormal behavior, and nightmares, were significantly frequently reported in children and in adults in both the Dutch and the global database. The RORs found in these adverse events were high, pointing to a strong relationship. In addition, although Aldea Perona et al. have argued that more neuropsychiatric symptoms were reported more frequently in children compared to adults (Aldea Perona et al. 2016), we cannot confirm this.

In a recent Spanish study, 24 patients (17 children and seven adults) reported nightmares after montelukast. In 18 patients, the nightmares appeared within the first week of treatment. In 21 cases, the nightmares rapidly resolved after montelukast had been discontinued (Cereza et al. 2012). The relatively high ROR indicates a strong statistical relation between montelukast and nightmares. This was true for both the Dutch and worldwide population. Although nightmares are often transient in children, they can be frightening for both child and parents and can influence school performance. In adults, sleep disorders can lead to potential dangerous situations in traffic or working with machines (Levin and Nielsen 2007; Simor et al. 2012). This means that the clinician must discuss the possibility of these adverse events with the patient and parents.

As a probable mechanism for the development of neuropsychiatric symptoms, it has been postulated that montelukast causes a higher blood‐brain permeability and inhibits the production of neurotransmitters such as serotonin and noradrenalin. Yet, human studies have revealed that the brain does not express leukotriene receptors (Singh et al. 2013) and that montelukast may even cause an inhibition of the blood‐brain barrier permeability (Biber et al. 2009). A reason for the higher incidence of agitation in children can be that children have more energy because their symptoms of asthma and/or allergic rhinitis are being tempered by montelukast and parents may interpret this as abnormal behavior or aggression (de Vries and van Hunsel 2016). As of yet, no pathophysiologic explanation is found.

It has been established that asthma symptoms are associated with depression and a lower quality of life (Goldney et al. 2003). This means that in some cases, the adverse effect is not a result of the drug but merely a result of unresolved asthma. Further research is required to reveal the mechanism for the higher incidence of neuropsychiatric symptoms in patients using montelukast in comparison with other medications.

The strength of this study is that the study material consisted of all pediatric and adult ADRs reports on montelukast located in both the database of the Netherlands Pharmacovigilance Center Lareb and the WHO Global ICSR database, VigiBase^®^. As far as we are aware, this is the first study that reports both ADRs in children and adults extracted from two large databases.

However, underreporting is a limitation of using a system of voluntary spontaneous reporting. This entails that the true occurrence of ADRs associated with montelukast cannot be extrapolated from these data. On the other hand, because reporting is voluntary, it will only occur when patients, parents, or professionals suspect a correlation. Moreover, a voluntary reporting system provides early warnings of drug‐related harm. Another limitation of voluntary reporting data is that the causality of the reported ADRs is not always certain.

## Conclusion

This article offers a comprehensive overview of the safety of montelukast in clinical practice. Serious ADRs include allergic reactions and chest pain. Although the relation between allergic granulomatous angiitis and montelukast is not elucidated, the prescribing physicians should be alert for signs and symptoms of this rare disease. Severe neuropsychiatric symptoms can occur after montelukast in both adults and children for whom montelukast was prescribed; especially nightmares may occur soon after starting montelukast.

## Author's Contributions

Study conception and design: MH, FH, TV; Data acquisition: FH; Data analysis: MH, FH, TV; Data interpretation: MH, FH, TV; Manuscript drafting and revising: MH, FH, TV.

## Disclosure

The authors report no conflicts of interest related to the manuscript.
